# Effect of behavior change communication through the health development army on birth weight of newborns in Ambo district, Ethiopia: a cluster randomized controlled community trial

**DOI:** 10.1186/s12905-024-03009-y

**Published:** 2024-03-26

**Authors:** Mitsiwat Abebe Gebremichael, Tefera Belachew Lema

**Affiliations:** 1 Department of Public Health, College of Health Sciences and Referral Hospital, Ambo University, Ambo, Ethiopia; 2 Department of Nutrition and Dietetics, Faculty of Public health, Jimma University, Jimma, Ethiopia; 3 Ambo University, P. O. Box 19, Ambo, Ethiopia

**Keywords:** Birthweight, Nutrition and health behavior change communication, Health development army, Pregnant women, Ambo district

## Abstract

**Background:**

Poor behavior change communication on maternal nutrition and health throughout pregnancy is thought to be to blame for Ethiopia’s high rate of low birthweight babies, and this has implications for neonatal morbidity and mortality. The effect of behavior change communication on birth weight in the study district was not examined. This study was to determine whether improving neonatal birthweight using nutrition and health behavior change communication (NHBCC) interventions was successful.

**Methods:**

A cluster randomized controlled trial was conducted in the Ambo district of Ethiopia from May 5, 2018–January 30, 2019. At the beginning of the study, 385 women in the 24 intervention groups and 385 women in the 24 control groups were recruited. In the intervention group, health development armies delivered the NHBCC core message every two weeks for four months by grouping pregnant women in specific clusters. Pregnant women in the control group received the routine treatment offered by the healthcare system during their ANC visits. Within 24 h of birth, the birthweights of 302 and 292 neonates in the intervention and control groups, respectively, were measured at the end point of the study. A binary generalized linear model analysis was employed.

**Result:**

The control group had a larger absolute risk of neonates with low birthweight (0.188 vs. 0.079, *p* < 0.001) than the intervention group. Pregnant women in the intervention group had an absolute risk difference of 10.9% for low birthweight. Pregnant women who received the intervention were 62% less likely to have low-risk birthweight compared to pregnant women who were in the control group (ARR = 0.381, 95% CI: 0.271–0.737).

**Conclusion:**

Nutrition and health behavior change Communication by health development armies improves birthweight. The findings demonstrated that to improve birthweight, NHBCC must be administered to pregnant women in groups via health development armies in their communities.

**Trial registration number:**

PACTR201805003366358.

**Supplementary Information:**

The online version contains supplementary material available at 10.1186/s12905-024-03009-y.

## Introduction

Low birthweight (LBW) is defined by the World Health Organization (WHO) as a newborn weighing less than 2500 g at birth, regardless of gestational age [1]. Preterm birth or intrauterine growth restriction (IUGR) are two possible causes [2]. Low birthweight is a worldwide public health issue. Around 15% of infants worldwide are born with LBW, resulting in over 20.5 million LBW babies each year. Almost all of these babies are born in low- and middle-income nations [3]. LBW affects 28% of all newborns in South Asia. LBW rates are estimated to be 13% in sub-Saharan Africa and 9% in Latin America and the Caribbean [4]. Because nearly half of all newborns are not weighed at delivery, the frequency of LBW is likely to be underestimated [4].

The prevalence of LBW in Ethiopia ranged from 7.8% [5] in Jimma, 14.6% [6] in Tigray, 18% [7] in the Kembata-Tembaro Zone and 22.2% [8] in the Amhara region of northern Ethiopia. According to a cluster randomized controlled trial conducted in West Gojam Zone, Ethiopia, nurses were counseled for the intervention group and routine nutrition education was given by the health system for the control group, and a higher proportion of newborns in the control group (14.7% vs. 6.4%, *p* = 0.002) had low birth weight than those in the intervention arm, which indicates the intervention reduced the prevalence of LBW [9].

According to an Ethiopian demographic and health survey (EDHS) study, the prevalence of LBW has risen from 11% in 2011 to 13% in 2016 [8]. According to a systematic review and meta-analysis conducted in Ethiopia, the national-pooled prevalence of low birth weight was 14.1% [10]. Furthermore, the frequency of LBW in the Oromia region, where the study was conducted, was 13% [11].

Birthweight is a powerful predictor of infant growth and survival. Infants born with a low birthweight face severe disadvantages and have extremely low survival rates [12]. LBW is a major underlying cause of infant and childhood mortality and morbidity [13]. In addition, findings also revealed that there is an association between LBW and increased risk for many chronic noncommunicable diseases in later life, such as diabetes mellitus type 2, cardiovascular diseases, hypertension, and cancer [14, 15]. Similarly, LBW infants will have different health problems like growth retardation, infectious diseases, and developmental delay, which may occur during infancy, childhood, and adult life [16].

Globally, WHO targets a 30% reduction in LBW between 2012 and 2025. However, the progress has been slower (1% worldwide yearly LBW reduction) from 2010 to 2015, compared to the 3% yearly target. Estimates by the WHO show that more than 20 million newborns globally suffered from LBW in 2015 [17].

There were different factors associated with low birthweight; among these were rural residence, household food insecurity, having an unemployed mother, maternal education, gender of the babies, preterm birth, caesarian delivery, first child order, having an unintended pregnancy, a lack of antenatal care visits, a birth interval less than 2 years, a previous history of having a LBW baby, pregnancy complications, maternal undernutrition, a lack of nutrition counseling, not taking an additional meal during pregnancy, and not taking an iron supplement [18–24].

Poor nutritional status and health, as well as inadequate food intake during pregnancy, have an impact not only on women’s health but also on birthweight and infant development [2]. Suboptimal nutrition and health practices due to the wrong perception in combination with environmental, sociodemographic, and economic factors and infections are common causes of low birthweight [22, 23, 25, 26].

Ethiopian pregnant women consumed less than the recommended amounts of several key nutrients, resulting in low birthweight and other negative birth outcomes [27]. The minimum dietary diversity among pregnant women in Ethiopia was low [28]. Below 50% (i.e., 47%) of pregnant women met the minimum dietary diversity in Ethiopia at the national level [29].

Since the prevalence of low birthweight is very high in Ethiopia, healthcare professionals can expect further research to lead to the development of an intervention or package of interventions that will impact the rates of low birthweight infants [8, 30].

Nutrition and health Behavior change communication (NHBCC) to improve health and caring practices is an integral component of efforts to improve maternal, newborn, and child health (MNCH) [31].

It has been realized that adequate information and positive attitudes alone are insufficient to motivate individuals and communities to take preventive action. A behavior change is required, which is closely tied to a change in illicit behaviors. Therefore, the messaging should be such that it emphasizes changing one’s behavior. Only professionally crafted, client-centered, benefit-oriented, service-linked, and research-based (NHBCC) makes it possible [32]. This is also supported by the health behavior change communication theory, the “Integrative Model of Behavioral Prediction,” which states that a person’s strong desire to perform a behavior, coupled with the necessary skills and abilities to perform it in the presence of a favorable environment, results in the desired behavior change [33].

The Health Development Army (HDA) is a group of organized women who represent the six nearest households (five members and one leader) in groups of five (1–5 networks) and larger groups, usually 30 households, known as women’s development groups (WDG). It was first introduced in Ethiopia in 2010. They are very supportive of pregnant women, encouraging them to give birth at health facilities with competent birth attendants [34].

HDAs are volunteers who can enhance access to primary health care (PHC) in Ethiopia and complement the work of Health Extension Workers (HEWs) [35]; there has been a considerable improvement in mother and child health and service usage since HDAs were introduced to the country; and they are also more connected to the community and are seen as a role model by women [36–39].

**Fig. 1:**
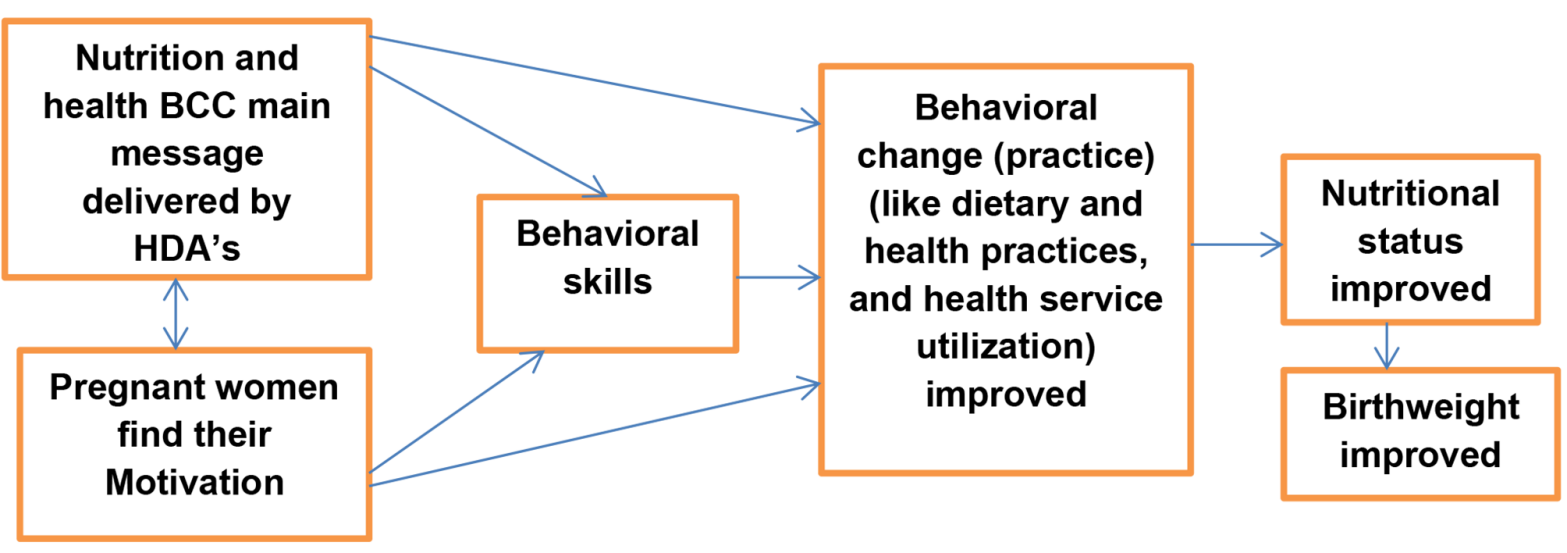
Diagrammatic presentations showing how behavioral change communication improves birth weight. (Adapted from the information-motivation-behavioral skills model Fisher & Fisher, 1992)

In summary, HDAs provide the main message (information) for study participants about the nutrition and health benefits, assisting them in finding their motivation, or increasing their objective behavioral skills or perceived self-efficacy could help them change their behavior to improve neonatal birthweight, as illustrated by the figure below (Fig. 1).

As far as we are aware, there has been no study done on the effect of NHBCC via the health development armies on the birth weight of newborns. Thus, this research aimed to evaluate the effect of NHBCC via the health development armies on the birthweight of neonates in the Ambo district.

## Research hypothesis

Nutrition and health behavior change Communication through the health development army can help improve the nutritional and health practices of pregnant women (primary hypothesis) [40].

Implementing preventive measures to lower low birthweight is crucial due to the high prevalence of LBW associated with insufficient dietary intake and other complications during pregnancy [41, 42]. Additionally, earlier studies hypothesized that improving prenatal counseling and education could lower the incidence of LBW [2, 9, 41, 43]. The effect of behavior change communication on neonatal birth weight, however, was not well understood. Therefore, this RCT was designed to determine whether NHBCC, through the health development armies during pregnancy, affected the birth weight of newborns (secondary hypothesis).

## Methods and materials

### Study design, study period and setting

A parallel cluster randomized controlled community trial (CRCCT) with a 1:1 allocation ratio was conducted from May 5, 2018–January 30, 2019, among pregnant women in the Ambo district of West Shoa Zone, Ethiopia. The researchers used sub kebeles (locally known as “Got”) as clusters in the trial. The Ambo district was chosen for the trial because low birthweight was a public health problem that required attention. The data were obtained from the district health office and other researchers [44]. This scenario drew the attention of the researchers to the decision to conduct this trial in these settings. One advantage of RCT is that it provides a very strong response to the causality debate, enabling authors and program implementers to be certain that the results they are seeing are solely the result of the intervention. A detailed explanation of the study area has been described in previously published work [40].

### Sample size determination

**Fig. 2:**
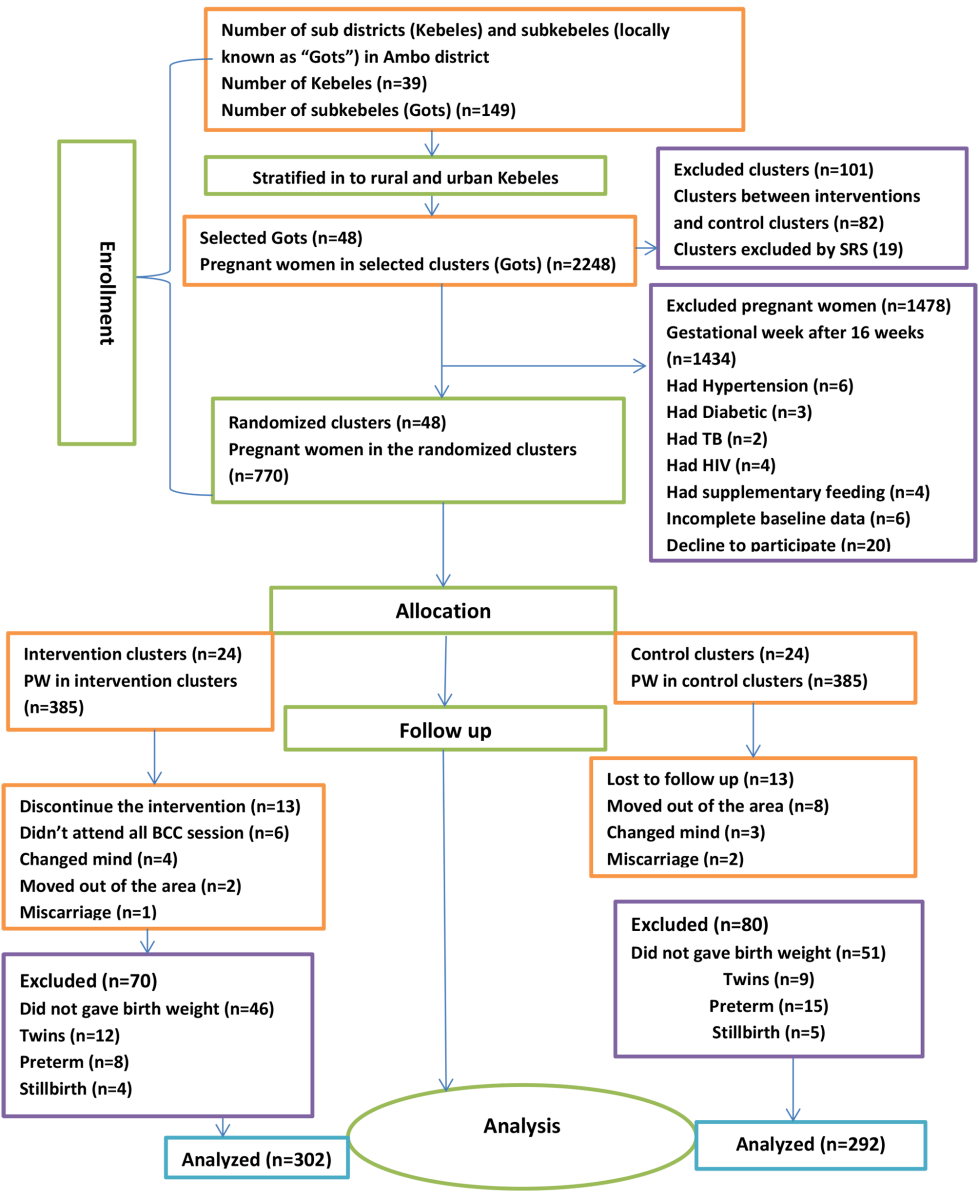
This diagram depicts the flow of study participants through the trial according to the criteria recommended in the CONSORT guideline

The sample size was calculated using the G Power 3.1.9.2 program with a power of 80% for Fisher’s exact test and a precision of 5%. According to a cluster randomized controlled trial conducted in the West Gojam Zone, Amhara region, the prevalence of LBW among exposed pregnant women (p1) 62% was used [45], with effect size (h) of 0.3 and with the allocation ratio of the intervention to control group (N2/N1) of 1, a prevalence (p2) 14.7% was obtained. The final sample size was 218 pregnant women per arm. Because cluster randomization was used, the calculated sample size was multiplied by the design effect of 1.5 due to cluster sampling, and a 10% loss to follow-up was considered, resulting in 360 pregnant women in the intervention group and 360 pregnant women in the control group who met the inclusion criteria. However, the sample size determined using G power for the first outcome of this trial (optimal nutrition and health practice) gave the largest sample sizes (385 women in the intervention group and 385 women in the control group), which were included in the trial [40]. Due to the problems reported in Fig. 2, the actual data were collected from 302 women in the intervention group and 292 women in the control group.

### Study population and eligibility criteria

The study population was pregnant women aged 18–49 years, pregnant women before 16 weeks of gestation, and permanent residents (who lived in the study area for more than six months) of the Ambo district in the selected clusters (Gots) of kebeles (the smallest administrative units). Pregnant women who were mentally ill and unable to speak or hear were excluded. Full details regarding the study population and eligibility criteria are described elsewhere [40].

### Recruitment of participants

A cluster sampling technique was used to select study participants. The district’s total number of kebele was stratified into rural and urban areas. Simple random sampling (SRS) with the lottery method was used to select 12 kebeles (2 urban and 10 rural) from the existing 39 kebeles (6 urban and 33 rural). Each kebele has its clusters (gots), which have been pre-determined by government bodies and are grouped into 149 distinct clusters. A proportionate stratified sampling strategy was employed to obtain clusters from each kebele.

The district’s non-adjacent clusters from each kebele were first identified. Then, the ones that were somewhat far apart from one another were selected and included in the study. Although it is challenging to show the distance in figures between included clusters, we left at least one cluster in between the clusters that we used for our research as a buffer zone. So, based on that, 48 clusters were chosen at random from the total number of clusters available in the district. As a result, this study included 24 clusters per arm. Eligible pregnant women were screened by the kebele’s health extension workers (HEWs) using a family folder prepared, as well as inquiring about the first date of their last menstrual cycle and confirming pregnancy using a pregnancy test, and the details are mentioned somewhere else [40].

### Randomization and intervention allocation

#### Random selection of clusters

The units of randomization in our study were the clusters, also referred to locally as “gots.” Each cluster received a unique cluster code. The 48 clusters were then divided into two blocks of size 4 by the alphabetical order in which they appeared. Using sealed lots from each of the six possible permutations inside each block, the author selected at random the cluster randomization sequence for each block. Each block cluster was randomly assigned to the intervention and control arms in the order indicated by the block’s selected permutation for the stratum. To maintain a 1:1 random allocation ratio, we formed 24 clusters for the intervention arm and 24 clusters for the control arm. All eligible pregnant women were enrolled in the same arm (either the intervention or control arm) in one cluster. Between the intervention and control clusters, buffer zones were left to minimize information contamination. There were typically 12–17 pregnant women in each cluster.

### Concealment

The nature of the intervention prevents allocation concealment. Both the intervention implementers (i.e., the HDAs as a team) and the pregnant women in the intervention clusters are aware of the intervention. Field supervisors were unaware of the outcome, and lastly, data collectors were blinded to the intervention. Additionally, by coding the two groups, the data entry clerk was rendered blind.

### Intervention

#### Part 1: Training of health development armies (HDAs)

The researchers used health extension workers to recruit Health Development Armies (HDAs). HDAs were selected from the intervention clusters. After recruitment, HDAs received a one-week training using an intervention protocol developed by the investigators for pregnant women based on the Essential Nutrition Action Framework, a framework for promoting maternal nutrition developed by the Manoff Group for developing countries, and making a balanced plate for pregnant women in Bangladesh [46–48].

The training comprised both theoretical and practical demonstrations. During the training of HDAs, role-playing, group meal preparation, and mock NHBCC sessions were held. The researchers developed a key assessment checklist that included theory and skill to lower variability among HDAs. The knowledge and skill levels of the Health Development Armies were evaluated before and after training using knowledge assessment tests as well as practical evaluations. To make sure that everyone was performing at a similar level after the training, the authors administered standardization tests.

#### Part 2: Group training of mothers by HDAs

The NHBCC messages were delivered to pregnant women within the clusters selected as intervention clusters. The intervention took place once every two weeks on non-working days. Each NHBCC session took 1:00–1:30 h. The researchers also supervised the training activities once every two weeks. The intervention lasted for four months. One HDA was responsible for one cluster. Full details of the intervention are found elsewhere [40].

The following health and nutrition messages are part of the intervention’s main messages: increasing meal frequency and portion size with gestational age, consuming a diversified diet, using iodized salt with appropriate utilization; not using foods like tea and/or coffee with meals; reduction of alcohol consumption; not avoiding important foods during pregnancy (associated with taboos); and taking iron or folate during pregnancy for at least three months, sleep under an insecticide-treated bed net, keep clean the environment, keep personal and food hygiene, reduce heavy workload and take rest, and make autonomous decisions, the need of family support during pregnancy, the use of health care services, etc. The consequences of not practicing these particular messages were also covered at each NHBCC session.

Each NHBCC session included an assessment of participant knowledge and attitudes toward optimal health and nutrition. NHBCC messages were then delivered by the gaps that were found. A message about food choices focused on easily accessible, socially acceptable, and reasonably priced meals.

HDAs received brochures and posters with self-explanatory pictures to show pregnant women in addition to the NHBCC messages. Leaflets with important messages were also written in the local languages and given to pregnant women. If a woman couldn’t read, it was advised that someone read the leaflet from her home or the neighborhood.

Pregnant women in the intervention group received six NHBCC sessions. The HDAs had attendance sheets in each session to keep track of and record participants’ adherence to the NHBCC sessions. The HDAs were given written teaching materials, a schedule, and topics to cover during every interaction (**Additional File 1**).

A practical demonstration included meal preparation, showing samples of iron and folate supplements, showing the type of iodized salt used and the timing of adding iodized salt while cooking, how to use an insecticide-treated bed net and personal and food hygiene techniques. To show how meals are prepared, participants were encouraged to share food from their homes. Pregnant women actively recognized the food types and preparation techniques they should follow based on this visual display.

#### Part 3: Home visits

Each HDA made a total of six home visits to pregnant women that sought to influence the behavior of pregnant women and their families (every two weeks). Each pregnant woman received individualized counseling and support during each home visit, which also served to reinforce the adoption of the practices she had been practicing during the group training sessions according to the protocols (Table 1).

**Table 1:** Nutrition and Health BCC key messages in the intervention clusters

No.	Key messages	BCC techniques applied	Supportive intervention
1	**Quantity-related messages**		Showed posters and brochures to the group and gave leaflets with key messages to a woman
1.1	Increase meal frequency (at least one extra diet)	Nutrition and health education	
1.2	Increase portion size with gestational age)	Counseling and nutrition and health education	
1.3	Avoidance or reduction of sharing food with others	Counseling	
2	**Quality-related messages**		
2.1	Consumption of fruits, vegetables, and animal products	Counseling, nutrition and health education	
2.2	Decrease consumption of iron-inhibiting foods, such as tea (coffee), with meals.	Counseling	
3	**Micronutrient intake**-**related messages**		
3.1	Take daily supplements of iron and folic acid during pregnancy for at least 3 months. Exhibiting samples of iron and folate tablets.	Nutrition and health education, and demonstration	
3.2	Use iodized salt for the whole family with appropriate usage. Exhibiting samples of iodized salt.	Nutrition and health education, and demonstration	
4	**Disease prevention and treatment practice (high priority for malaria and worms) related messages**		
4.1	Pregnant women with a fever need to be taken to a health facility for immediate treatment.	Counseling	
4.2	Keeping the environment clean	Counseling	
4.3	Wash hands with soap during key contact moments (critical occasions) and drink treated water.	Nutrition and health education and demonstration	
4.4	Keeping food and food Containers clean.	Nutrition and health education, and demonstration	
4.5	Sleep under an insecticide-treated bed net.	Demonstration	
5	**Supportive lifestyle and care-related messages**		
5.1	Had an appropriate workload and rest during pregnancy.	Counseling	
5.2	Improve decision-making power in food and health.	Counseling	
5.3	Get support from family or other individuals during pregnancy.	Counseling	
5.4	Utilization of health care services (ANC follow-up, plan to deliver at a health institution, and PNC follow-up)	Counseling	
5.5	Birth preparedness and complication readiness	Counseling	

Pregnant women in the intervention group received NHBCC intervention via HDAs for a period of four months from July to October 2018.

Pregnant women in the control group were exposed to the standard care provided by the health care system during their ANC visit and any intervention at the community level by Health Extension workers. This service was available to pregnant women in both the control and intervention groups. They received the same evaluations and were observed for the same amount of time as the intervention group.

### Data collection and measurement

A semi-structured and pretested English-version questionnaire was used to collect the data. The questionnaire was translated into two local languages and then back into English by language professionals to ensure consistency. Before the actual data collection, many skip patterns were fixed, and the questionnaire was pretested in Ginchi town, which is adjacent to the Ambo district, for any ambiguity, length, completeness, consistency, and acceptability.

To collect data, eight diploma nurses were recruited. The data collectors received training on the study’s purpose and relevance, data confidentiality, respondent rights, informed consent, and interview techniques. Face-to-face interviews at the participants’ residences were used to collect data. Four supervisors with BSc degrees in nursing and the main investigator on the spot examined the completed questionnaires daily for consistency and completeness.

The questionnaire has four parts. Part one of the questionnaire consists of socio-demographic and economic characteristics; Part two of the questionnaire consists of maternal characteristics; Part three of the questionnaire consists of knowledge, attitudes, and practices about nutrition and health; Part four of the questionnaire consists of nutritional status; and Part five of the questionnaire consists of the birthweight of neonates.

Data on socio-demographic, economic, and maternal characteristics were gathered at baseline. Before and after the trial, data on nutrition and health knowledge, attitude, dietary practice, nutritional status, and women’s weight were obtained.

Full details of the data collection techniques for the household wealth index, women’s decision-making power, food security status, salt iodization status, and knowledge and attitudes on nutrition and health are found elsewhere [40].

The dietary practice was assessed using a semi-structured questionnaire developed and modified from the Food and Nutrition Technical Assistance III Project (FANTA) [49]. This tool records in-depth information on all foods and drinks consumed in the previous 24 h, including any snacks, with an estimation of the portion size from sunrise to sunrise. Interviewers also probed for any food types that participants may have forgotten. Based on the nutrients in each food item, we combined all foods consumed into 10 food groups [50]. We collected data on dietary diversity over four nonconsecutive days longitudinally [46]. The dietary diversity score was ranked into terciles, with the highest tercile used to indicate a “high” dietary diversity score and the two lower terciles taken to indicate a “low” dietary diversity score [51].

The frequency of each animal-source food consumed by the women over four nonconsecutive days was used to assess their utilization of animal-source food (ASF). Finally, terciles were formed by dividing the frequency of ASF consumption [52].

Dietary practices were assessed using the dietary diversity score, consumption of animal-source foods, and increased meals (both in frequency and amount). Women who had increased meal quantities had higher dietary diversity scores and had higher ASF scores were considered to have optimal dietary practices, whereas women who didn’t increase meal quantities had lower dietary diversity scores, or had lower ASF scores were considered to have suboptimal dietary practices [53].

The primary outcome variable (i.e., nutrition and health practice) was assessed using 14 questions that were used to assess nutrition and health practice and included questions about dietary quantity, dietary quality, micronutrient intake, disease prevention and treatment, and supportive lifestyle and care [47, 48]. The details of the data collection and measurement related to nutrition and health practices were available somewhere [40].

### Mid upper arm circumference measurements

The nutritional status of the pregnant women was assessed by measuring their mid-upper arm circumference (MUAC). A non-stretchable MUAC tape was used to measure MUAC. A MUAC of the upper left arm was taken with no clothing on the arm. The left arm was chosen because it demonstrates malnutrition, whereas the right arm, which is commonly employed, demonstrates lean muscle mass gained via work [54].

During the procedure, the midpoint of the upper arm was located by flexing the women’s elbows to 90^0^ with the palm pointing upwards. Then the distance from the acromion to the olecranon processes was measured, and the midpoint was marked with ink. Finally, the measuring tape was placed snugly around the arm at the midpoint mark while hanging the arm freely, palm facing towards the thigh. On the same day, each study subject had two measurements taken and read to the nearest 0.1 cm. Women with MUAC > = 23 cm were considered normal-nourished, whereas participants with MUAC < 23 cm were considered undernourished [55, 56].

The birthweight of the neonates was the secondary outcome of this study. Health Development Armies notify whether the woman gives birth or not within 24 h. Data collectors visit the mother and baby and weigh the baby. Data were gathered at the residence for those who gave birth at home and for those who gave birth at a health facility and returned to their home within 24 h of giving birth. However, birthweight information was gathered from the health facility for those who gave birth at a health facility within the study district and planned to stay longer than 24 h because of various problems or complications after consulting with a healthcare professional.

The weight of the baby was measured on balanced digital Seca scales, which were read to the nearest 100 g [57]. Before each measurement, the scales were calibrated with a known-weight object. Furthermore, before weighing each newborn, the readings on each scale were adjusted to zero. Low birthweight is defined as a birthweight of less than 2500 g. The women wore light clothing while being weighed. By subtracting the baseline weight from the end-line weight, gestational weight gain was estimated. The authors kept track of when the newborns were weighed and found no significant differences in birth weight between the intervention and control groups.

Baseline data on dietary practices and nutritional status were collected from pregnant women (*n* = 770) from June 1–21, 2018; endline data on dietary practices and nutritional status were collected from pregnant women (*n* = 744) in December 2019; and data on LBW were collected from pregnant women (*n* = 594) in January 2019.

### Data processing and analysis

Multiple births, preterm deliveries, stillbirths, and pregnant women who did not give birth to a healthy baby were excluded from the analysis. Before entering the data, data were manually checked for completeness and consistency. Then it was entered into EPI data version 3.1 and exported to SPSS for Windows version 23 for cleaning and analysis.

Since cluster randomization was used, a generalized linear mixed model was fitted to include both cluster-level and individual-level variables. The intercept-only model estimates the intercept as 0.63 (the average newborn’s birthweight across all clusters was 0.63 but wasn’t statistically significant (*p* = 0.215)). The intra-cluster correlation coefficient was also closer to zero (0.038). This implied that 96.2% of the newborns’ birthweight was explained by individual-level variables. As a result, we didn’t use generalized linear mixed model analyses for cluster-level variables. The non-significant variation in newborn birth weight at the cluster level could be due to the sample size calculation accounting for the design effect of 1.5, which increased the sample size computed. Therefore, bivariable and multivariable generalized linear model analyses were used to assess predictors at individual levels for the two reasons described above.

For continuous variables, descriptive statistics like mean and standard deviation were first determined, and for categorical data, frequency and percentage. To compare the baseline characteristics of the intervention and control groups, a chi-square test was used. Similarly, using independent sample t-tests, the mean and standard deviation, as well as their p-value, were used to compare newborns’ birth weight, gestational age in weeks, and gestational weight gain of pregnant women between the intervention and control groups. In addition, proportions with their p-values were conducted to compare newborns’ birth weight, dietary practices (before and after intervention), and the MUAC of pregnant women (before and after intervention).

Multicollinearity was checked using variance inflation factors (VIF), and there was no multicollinearity between independent variables. The study of effect measures was done using the RR with 95% CI and p-values. A bivariate generalized linear analysis was performed between the birthweight of neonates and associated factors one at a time. Their relative risks (RR) with 95% confidence intervals (CI) were obtained. Factors that were significantly associated with the birthweight of neonates at a p-value < 0.25 in the bivariable generalized linear analysis were entered into the multivariable generalized linear analysis (i.e., the final model). A p-value of < 0.05 and 95% CI were used to assess statistical significance. Statistical analysis was conducted on the intention-to-treat analysis.

### Data quality control

The Health Development Armies trained together for one week. Additionally, three days of training were provided for data collectors and supervisors. The HDAs were supervised by supervisors and investigators every two weeks and discussed issues that came up during BCC sessions and offered possible solutions. The lengths of contact within each intervention group were similar, and pregnant women in each cluster attended the same number and frequency of NHBCC sessions. In a personal training diary (attendance sheet), the HDA monitored and noted participants’ adherence to the BCC sessions. Before the start of the experiment, the intervention process was pretested.

The entire scale of the instrument had a Cronbach’s alpha value of > 0.7 for the knowledge, attitude, and practice parts. To help respondents’ comprehension, the questionnaire was pretested and translated into local languages. Supervisors and the main investigator closely monitored the data collection process. Every day, completed questionnaires were checked for accuracy and updated with any incorrect or missing data. Field supervisors looked at 5% of the data at random and informed them about a possible measurement problem [46].

## Results

### Baseline socio demographic and maternal characteristics of pregnant women

At baseline, a total of 770 pregnant women (385 in the control group and 385 in the intervention group) were recruited. Overall, birthweights of 594 (77.1%) neonates (302 from the intervention group, 292 from the control group) were measured within 24 h after birth and included in the analysis. However, 176 (22.9%) neonates were not included in the analysis.

The major reasons for neonates not being included in the final analysis were that they did not give birthweight 46 (11.9%) from the intervention group and 51 (13.2%) from the control groups, for a total of 97 (12.6%), gave twins birth 12 (3.1%) from the intervention group and 9 (2.3%) from the control groups, in total 21 (2.7%), gave preterm birth 8 (2.1%) from the intervention group and 15 (3.9%) from the control groups, in total 23 (3.0%), gave stillbirth 4 (1.0%) from the intervention group and 5 (1.3%) from the control groups, in total 9 (1.2%), discontinued the intervention 13 (3.4%) from the intervention group and lost to follow-up 13 (3.4%) due to the mentioned reason in Fig. 2.

At baseline, there were no significant differences in all sociodemographic and obstetric characteristics between the intervention and control groups (*p* > 0.05) (Table 2).

**Table 2:** Baseline socio-demographic and obstetric characteristics of pregnant women in control and intervention groups, Ambo district, Ethiopia, 2018

Variable	Category	Intervention group (*n* = 302) Frequency (%)	Control group (*n* = 292) Frequency (%)	p
Residence	Rural	240(79.5)	244(83.6)	0.199
	Urban	62(20.5)	48(16.4)	
Age of the respondent	18–24 Years	79(26.2)	66(22.6)	0.302
	25–34 Years	203(67.2)	198(67.8)	
	> 35 Years	20(6.6)	28(9.6)	
Marital status	Married	285(94.4)	280(95.9)	0.390
	Widowed/Divorced/non married partner	17(5.6)	12(4.1)	
Religion	Orthodox	133(44.0)	143(49.0)	0.06
	Protestant	130(43.0)	126(43.2)	
	Catholic/Muslim	21(7.0)	12(4.1)	
	Wakefeta	18(6.0)	11(3.8)	
Respondents’ Occupation	Employed	15(5.0)	16(5.5)	0.621
	Housewives/ Daily laborers	250(82.8)	238(81.5)	
	Merchants	19(6.3)	14(4.8)	
	Farmers	18(6.0)	24(8.2)	
Husband Occupation	Employed	28(9.3)	36(12.3)	0.652
	Merchants	35(11.6)	32(11.0)	
	Farmers	182(60.3)	179(61.3)	
	Daily laborers	33(10.9)	25(8.6)	
	Private workers	24(7.9)	20(6.8)	
Women educational status	No formal education	115(38.1)	108(37.0)	0.607
	1–4 Grade	68(22.5)	75(25.7)	
	5–8 Grade	76(25.2)	67(22.9)	
	9–12 Grade	35(11.6)	29(9.9)	
	Diploma and higher	8(2.6)	13(4.5)	
Husband educational status	No formal education	89(29.5)	83(28.4)	0.233
	1–4 Grade	53(17.5)	56(19.2)	
	5–8 Grade	85(28.1)	63(21.6)	
	9–12 Grade	58(19.2)	64(21.9)	
	Diploma and higher	17(5.6)	26(8.9)	
Household size	1–3 household size	98(32.5)	81(27.7)	0.453
	4–5 household size	127(42.1)	130(44.5)	
	> 5 household size	77(25.5)	81(27.7)	
Household wealth tertile	Low	91(30.1)	78(26.7)	0.607
	Medium	131(43.4)	129(44.2)	
	High	80(26.5)	85(29.1)	
Estimated time to reach health institution	< 30 min	65(21.5)	60(20.5)	0.596
	30–60 min	121(40.1)	108(37.0)	
	> 60 min	116(38.4)	124(42.5)	
Parity	<=one child	99(32.8)	90(30.8)	0.674
	2–4 Children	176(58.3)	170(58.2)	
	5 and above children	27(8.9)	32(11.0)	
Gravida	One	51(16.9)	43(14.7)	0.352
	2–4	187(61.9)	173(59.2)	
	5 and more	64(21.2)	76(26.0)	
Start of ANC	No	124(51.9)	115(48.1)	0.612
	Yes	178(50.1)	177(49.9)	

### Gestational weight of pregnant women in the intervention and control groups

At baseline, the weight of pregnant women between intervention and control was comparable (53.2 ± 4.99 vs. 52.56 ± 4.02, *p* > 0.05). At the end of the trial, the weight of women in the intervention group had improved (63.93 ± 7.06 vs. 61.88 ± 5.97, *p* < 0.001) compared to their counterparts.

### Birth weight, gestational weight gain and gestational age of pregnant women

The mean birth weight of neonates born in the intervention group was 3.03 kg (± 0.41) at the end of the study, compared to 2.73 kg (± 0.35) in the control group, indicating that the intervention group had a 0.3 kg higher birth weight. The intervention group’s mean gestational weight gain was 10.73 kg (± 2.07) compared to 9.32 kg (± 1.95) in the control group, indicating that the intervention group gained 1.41 kg more. The intervention and control groups had significantly different mean gestational ages at birth (mean ± SD = 38.6 (± 1.3) vs. 37.7 (± 1.5), *p* < 0.001) (Table 3).

**Table 3:** Comparison of birth weight, gestational weight gain and gestational age of pregnant women in control and intervention groups in Ambo district, Ethiopia, 2018

Variables	Intervention Mean (± SD) (Kg)	Control Mean (± SD) (Kg)	Difference Mean (SE) (Kg) (95% CI)		p
Birth weight(kg)	3.03((± 0.41)	2.73 kg (± 0.35)	0.3(0.03)	0.24–0.36	< 0.001
Gestational weight gain(kg)	10.73 kg(± 2.07)	9.32(± 1.95)	1.4(0.17)	1.08–1.73	< 0.001
Gestational age (week)	38.6(± 1.3)	37.7(± 1.5)	0.9(0.11)	0.67–1.10	< 0.001

### Birthweight, dietary practice and nutritional status of pregnant women

A higher proportion of the newborns in the control group had low birthweight than the intervention group (18.8% vs. 7.9%, *p* < 0.001). In the intervention group, there were two macrosomic babies (0.7%), whereas there were no macrosomic babies in the control group (Table 3). The intra-cluster correlation (ICC) coefficient was closer to zero (0.038).

The study participants’ dietary practices were comparable before the intervention (*p* = 0.732). At the end of the trial, the intervention group had a smaller percentage of women with poor dietary practices (36.4% vs. 63.6%, *p* < 0.001) than the control group (Table 4).

Before the intervention, the MUAC of the study subjects was comparable (*p* = 0.55). At the end of the trial, a lower proportion of women in the intervention group (13.2% vs. 26.7%, *p* < 0.001) had lower MUAC than their counterparts (Table 4).

**Table 4:** Comparison of the birthweight of neonates and nutritional status of pregnant women in Ambo district, Oromia, Ethiopia, 2018

Variables	Category	Intervention (n_1_ = 302)	Control (n_2_ = 292)	p
Birth weight	Low birth weight (< 2500 g)	24(7.9)	55(18.8)	< 0.001
	Normal birth weight (2500-4000 g)	276(91.4)	237(81.2)	
	Macrosomic (> 4000 g)	2(0.7)		
ICC		0.038		
Dietary practice before intervention	Suboptimal	222 (73.5)	211(72.3)	0.732
	Optimal	80(26.5)	81(27.7)	
Dietary practice after intervention	Suboptimal	110(36.4)	170(57.8)	< 0.001
	Optimal	192(63.6)	122(42.2)	
MUAC before intervention	< 23 cm	86(22.9)	93(24.8)	0.549
	> =23 cm	289(77.1)	282(75.2)	
MUAC after intervention	< 23 cm	40(13.2)	78(26.7)	< 0.001
	> =23 cm	262(86.8)	214(73.3)	

*Abbreviation* MUAC, mid-upper arm circumference

### Risk factors for birthweight at the endpoint of the study among pregnant women in the Ambo district

The control group had a larger absolute risk of neonates with low birthweight (0.188 vs. 0.079, *p* < 0.001) than the intervention group. Pregnant women in the intervention group had a relative risk and absolute risk difference of 0.381 and 10.9% for low birthweight, respectively.

Bivariable generalized linear model Analysis showed that there was an association between birthweight of newborns and study group, age, respondent educational status, knowledge of nutrition and health (endline), attitude towards nutrition and health (endline), dietary practice (endline), and MUAC of mothers (endline). Whereas, residence, religion, marital status, ethnicity, age at marriage, respondent occupation, household size, wealth status, food security status, number of previous pregnancies, number of previous births, gap duration between pregnancies, estimated time to reach a health institution, and timing newborn weight measurement (within the range of 24 h) had no association with the birthweight of newborns. However, study group, religion, dietary practice (endline), and MUAC measures (endline) were found to be significantly associated with the birthweight of the neonates in multivariable generalized linear model analysis (*p* < 0.05) (Table 5).

The study group (being in the intervention group) was found to have a significant association with the birthweight of the neonates. Pregnant women who received the intervention were 61.9% less likely to have the risk of low birthweight neonates compared to pregnant women who were in the control group (ARR = 0.381, 95% CI: 0.271–0.737). The study revealed that pregnant women who were orthodox in their religion had a 1.6 times higher risk of having low birthweight neonates than their counterparts (ARR = 1.648, 95% CI: 1.045–2.598). The study revealed that pregnant women who had optimal dietary practices were 63.5% less likely to have the risk of low birthweight neonates than their counterparts (ARR = 0.365, 95% CI: 0.253–0.688). The study also found that pregnant women who had MUAC > = 23 cm were 72.0% less likely to have the risk of low birthweight neonates than their counterparts (ARR = 0.28; 95% CI: 0.19–0.523) (Table 5).

**Table 5:** Bivariable and multivariable generalized linear model Analysis for factors associated with birthweight among newborns delivered in in Ambo district, Ethiopia, 2018

Variable	Category	Birth weight		CRR(95% CI)	ARR(95%CI)
		**Normal** **N (%)**	**LBW** **N (%)**		
Study group	Control	237(81.2)	55(18.8)	1	1
	Intervention	278(92.1)	24(7.9)	0.422(0.269–0.663)	0.381(0.271–0.737)*
Religion	Protestant	233(89.6)	27(10.4)	1	1
	Orthodox	227(83.8)	44(16.2)	1.563 (0.999–2.447)	1.648(1.045–2.598)*
	Others	55 (87.3)	8 (12.7)	1.223(0.584–2.561)	0.998(0.491–2.028)
Age of pregnant women	18–24 years	134(26.0)	9(11.4)	1	1
	25–34 years	342(66.4)	64(81.0)	2.505(1.280–4.901)	1.603(0.735–3.496)
	>=35 years	39(7.6)	6(7.6)	2.119(0.797–5.629)	1.269(0.404–3.983)
Age at marriage	< 18 years	104(20.2)	10(12.7)	1	1
	18–24 years	387(75.1)	63(79.7)	1.596(0.846–3.011)	1.300(0.511–3.311)
	> 24 years	24(4.7)	6(7.6)	2.280(0.901–5.772)	1.339(0.706–2.538)
Respondent educational status	No formal education	182(80.9)	43(19.1)	1	1
	Primary education	116(85.3)	20(14.7)	0.769(0.473–1.251)	0.875(0.516–1.485)
	Secondary and above education	217(93.1)	16(6.9)	0.359(0.209–0.619)	0.524(0.261–1.053)
Household size	<=2household	73(91.3)	7(8.8)	1	1
	3–4 household	192(86.1)	31(13.9)	1.589(0.729–3.464)	0.982(0.429–2.251)
	>=5 household	250(85.9)	41(14.1)	1.610(0.751–3.451)	0.635(0.254–1.588)
Gravidity	<=2 pregnancy	167(89.8)	19(10.2)	1	1
	3–4 pregnancy	235(86.1)	38(13.9)	1.363(0.812–2.288)	0.804(0.426–1.516)
	>=5 pregnancy	113(83.7)	22(16.3)	1.595(0.900-2.828)	1.121(0.508–2.472)
Gap duration between pregnancy	1st pregnancy	83(91.2)	8(8.8)	1	
	2nd pregnancy	180(85.7)	30(14.3)	1.625(0.775–3.406)	1.129(0.513–2.484)
	>=3rd pregnancy	252(86.0)	41(14.0)	1.592(0.775–3.270)	1.154(0.539–2.468)
Wealth status	Low	155(30.1)	17(21.5)	1	1
	Medium	229(44.5)	38(48.1)	1.440(0.840–2.468)	1.118(0.642–1.948)
	High	131(25.4)	24(30.4)	1.567(0.875–2.804)	1.371(0.757–2.484)
Estimated time to reach Health institution	< 30 min	116(90.6)	12(9.4)	1	1
	30–60 min	203(87.1)	30(12.9)	1.373(0.729–2.588)	1.487(0.779–2.839)
	>=60 min	196(84.1)	37(15.9)	1.694(0.916–3.131)	1.607(0.843–3.064)
Knowledge on nutrition and health	Poor Knowledge	260(50.5)	59(74.7)	1	1
	Good Knowledge	255(49.5)	20(25.3)	0.393(0.243–0.636)	0.638(0.351–1.158)
Attitude towards nutrition and health	Unfavorable Attitude	322(62.5)	59(74.7)	1	1
	Favorable Attitude	193(37.5)	20(25.3)	0.606(0.376–0.979)	1.379(0.782–2.431)
Nutrition and health Practice	Suboptimal	221(42.9)	59(74.7)	1	1
	Optimal	294(57.1)	20(25.3)	0.302(0.187–0.489)	0.365(0.253–0.688)*
MUAC of pregnant women	< 23 cm	74(14.4)	44(55.7)	1	1
	> =23 cm	441(85.6)	35(44.3)	0.197(0.133–0.293)	0.28(0.19–0.523)*

*Abbreviations*: N: Number ; CRR, crude relative risk; ARR, adjusted relative risk

*Significant at p-value < 0.05. Parameter estimates were adjusted for the tabulated variables

## Discussion

This trial has shown the effect of NHBCC through the Health Development Army on neonatal birthweight in Ambo District, Ethiopia. The results of this study support the NHBCC’s effectiveness in improving neonates’ birth weight. Women who received BCC on nutrition and health had better birthweights than women who did not receive BCC. The mean birth weight of newborns in the intervention group was higher by 0.3 kg than the control group. This finding is consistent with the studies done in Kenya and Bangladesh [58, 59]. Similarly, nutrition education for pregnant women is thought to influence nutrition-related knowledge and dietary behavior, pregnancy weight gain, and birthweight, according to a study by Zeng L. et al. [60].

Nutrition and health behavioral change communication interventions were successful in enhancing pregnant women’s dietary and health habits. This study is in agreement with a study among Indonesian pregnant women that found improved nutrition and dietary patterns in pregnant women using small groups and interactive approaches [61]. Similarly, women who received BCC on nutrition and health had a better nutritional status than women who did not receive BCC. This is congruent with the findings of Sharifirad GR. et al., found that nutrition interventions had a positive effect on improving nutritional status during pregnancy when compared to traditional training [42]. Through raised awareness and knowledge, nutrition and health practices and nutritional status were improved, and these can be expected to improve neonatal birthweight [43, 62].

One possible explanation for the difference between the intervention and control groups is that the intervention was founded on practical demonstration, a unique and intriguing behavior change communication strategy. Health development armies delivered the key messages of the intervention mentioned in the method section, strictly following the protocol. The following core interventional elements were successfully implemented by pregnant women at the end of the study period: consuming additional food as gestational age increased; using iodized salt; avoiding important foods (associated with taboos); taking iron and folate for at least three months during pregnancy; seeking medical attention from a facility if she fell ill (a priority for malaria and deworming); washing hands with soap at crucial times; a high workload; and taking days off [40]. The nutritional and health habits of pregnant women are significantly influenced by all of these variables. This has a significant impact on reducing newborn birthweight.

Generally, the possible explanation could be that women in the intervention group have received BCC about maternal diet, preventive actions, seeking treatment if sick, and the necessity of doing so based on the intervention protocol. Women’s understanding of the implications of bad eating, the benefits of following optimal dietary practices, and weight growth rose as a result of this sort of BCC [63]. As a result, gestational weight increases and birthweight are improved [58].

This intervention is simple to execute by existing community health workers (health development armies), and the pregnant women involved are engaged since it is participatory. It doesn’t necessitate any additional props or tools that aren’t already in their possession. Unlike nutrition education, such as that provided to controls, our approach is more visual and engaging, making it simpler for participants to grasp the information. Importantly, it does not necessitate food supplementation, making it highly scalable and more likely to be sustainable in resource-poor situations like Ethiopia. In contrast, health education provided by health professionals at health institutions during ANC visits or in the community emphasized the importance of women eating meals available at home by adding one extra meal to their regular diet. During the third trimester, one extra meal may not be adequate for all pregnant women, and the food provided at home may be undiversified. This form of generalized, non-specific teaching is unlikely to boost birthweights.

Another explanation might be that: (1) we conducted our intervention in their communities by grouping pregnant women; (2) health development armies visited the homes of each pregnant woman; this promoted communication and developed skills among the pregnant women; these, in turn, increased pregnant women’s self-confidence and efficacy; and these, in turn, led to behavior change.

The study’s findings have practical relevance for preventing low birthweight babies. The community-level BCC intervention should be promoted by the health development armies, monitored, and supported by health professionals using the frameworks created by the authors, as evidenced by the fact that the intervention group had significantly fewer newborns with LBW.

## Limitation

The study acknowledges the following limitations: Allocation concealment was not possible due to the nature of the intervention; however, neither the pregnant women in the intervention group nor the data collectors were aware of the research question.

Another weakness of this study is the failure to follow up. Despite the significant rate of failure to follow up, the baseline characteristics of the study participants were similar in both groups.

During the data collection period, pregnant women’s dietary and health-related practices were self-reported and could not be cross-checked, so memory and truthfulness in answering the questions are issues. The results could only be generalized to the study district.

## Conclusion

Nutrition and health behavior change communication (NHBCC) through community-level actors (health development armies) had a positive effect on improving the birthweight of neonates. As a result, through already-existing community institutions, this scalable nutrition and health behavior change communication intervention, with a focus on health development armies, may be scaled up and sustained with minimal investments. Before scaling up the intervention, policymakers should work on developing guidelines with a core message on optimal nutrition and health during pregnancy.

### Electronic supplementary material

Below is the link to the electronic supplementary material.

Supplementary Material 1

Supplementary Material 2

Supplementary Material 3

## Data Availability

All data generated or analysed during this study are included in this published article [and its supplementary information files].

## Abbreviations

ANC: Antenatal Care
ARR: Adjusted relative Risk
NHBCC: Nutrition and Health Behavior Change Communication
CRR: Crude relative Risk
CI: Confidence Interval
CONSORT: Consolidated Standards of Reporting Trials
CRCCT: Cluster Randomized Controlled Community Trial
DDS: Dietary Diversity Score
ASF: Animal Source Food
EDHS: Ethiopian Demographic and Health Survey
ENA: Essential Nutrition Action
HDA: Health Development Army
HEWs: Health Extension Workers
IUGR: Intrauterine growth restriction
LBW: Low birthweight
MNCH: maternal, newborn, and child health
MUAC: Mid-upper Arm Circumference
PACTR: Pan African Clinical Trial Registry
PHC: primary health care
PNC: Post natal care
PCA: principle components analysis
WDG: women’s development groups
WHO: World Health Organization
