# Supplementary material for: Effect of behavior change communication through the health development army on birth weight of newborns in Ambo district, Ethiopia: a cluster randomized controlled community trial
Source: BMC Womens Health. 2024 Mar 26;24:200. doi: 10.1186/s12905-024-03009-y (PMC10964566; doi:10.1186/s12905-024-03009-y)
Supplement: Supplementary file 3 — Supplementary Material 3 [file 12905_2024_3009_MOESM3_ESM.docx]

**Supporting documents**

**Additional file 1:** Intervention Protocol on the effect of behavior change communication through the health development army on optimal nutrition and health practices of pregnant women in West Shoa Zone, Oromia, Ethiopia: a cluster randomized controlled community trial

**Additional file 2:** CONSORT 2010 checklist of information to include when reporting a randomized trial
